# Fish as bioindicators for trace element pollution from two contrasting lakes in the Eastern Rift Valley, Kenya: spatial and temporal aspects

**DOI:** 10.1007/s11356-017-9518-z

**Published:** 2017-07-07

**Authors:** Christof Plessl, Elick O. Otachi, Wilfried Körner, Annemariè Avenant-Oldewage, Franz Jirsa

**Affiliations:** 1 Faculty of Chemistry, Institute of Inorganic Chemistry, University of Vienna, Althanstrasse 14, 1090 Vienna, Austria; 2 Biological Sciences Department, Egerton University, P. O. Box 536, Egerton, Kenya; 3 Department of Environmental Geosciences, University of Vienna, Althanstrasse 14, 1090 Vienna, Austria; 4 Department of Zoology, University of Johannesburg, P. O. Box 524, Auckland Park, 2006 South Africa

**Keywords:** Trace elements, Heavy metals, Lake Naivasha, Lake Turkana, *Tilapia zillii*, *Cyprinus carpio*

## Abstract

Lake Turkana and Lake Naivasha are two freshwater lakes in the Kenyan Rift Valley that differ significantly in water chemistry and anthropogenic influence: Lake Turkana is believed to be rather pristine and unpolluted, but a previous study has shown rather high levels of Li, Zn, and Cd in the migratory fish species *Hydrocynus forskahlii*, questioning this pristine status. Lake Naivasha is heavily influenced by agricultural activity in its catchment area and by direct water use, and high levels of metal pollutants have been reported in fish. This study presents the distribution of nine important trace elements in liver and muscle of the nonmigratory red belly tilapia *Tilapia zillii* from Lake Turkana and from Lake Naivasha (before and after a significant rise in water level due to as yet not fully understood reasons). In addition, trace element levels in the common carp *Cyprinus carpio* from Lake Naivasha are presented. Metal concentrations measured in the liver and muscle of *T. zillii* collected in Lake Turkana confirm the pristine status of the study site, but contrast with the results obtained for the migratory *H. forskahlii*. Comparing *T. zillii* from the two lakes reveals a clear difference in accumulation patterns between essential and nonessential trace elements: physiologically regulated essential elements are present in a very similar range in fish from both lakes, while levels of nonessential metals reflect short- or long-term exposure to those elements. The comparison of trace element concentrations in the fish samples from Lake Naivasha showed lower levels of most trace elements after the significant increase of the water level. This study demonstrates that fish are valuable bioindicators for evaluating trace element pollution even in contrasting lakes as long as the way-of-life habits of the species are taken into account.

## Introduction

Trace elements have long been of great interest in environmental monitoring. Although they are natural components of the aquatic environment and some of them, for example, Zn and Cu, are essential for most biota in certain concentrations, human activities have led to their remobilization in the environment. Exposure to elevated levels poses a threat to wildlife health in many regions of the world (Reisinger et al. 2009). The main anthropogenic sources for pollution are domestic and industrial emissions through waste effluents and emissions caused by mining, energy production, and agricultural activities (Förstner and Wittmann 1983). Four out of the six top contaminants in the world reported by Pure Earth (2015) belong to the class of trace elements, namely Pb, Hg, Cr, and Cd. Many of these elements such as Pb or Hg have long been known for their toxic properties, and therefore, at least in wealthier countries, enormous efforts have been taken to decrease their release into the environment (Järup 2003). Amongst others, the European Union has set maximum levels for Cd, Pb, Hg, and Ni as priority substances regarding aquatic environments in the directive on environmental quality standards in the field of water policy (European Union 2008). In many European countries, the criteria demanded in this law have already been met (European Environment Agency 2012). With regard to human food safety, the European Union added the ALARA (as low as reasonably achievable) principle for Pb—in addition to the maximum allowed levels in food for human consumption to their regulation—to further reduce Pb uptake (European Union 2006). Canada is working on a similar regulation (Health Canada 2011). However, millions of people’s health is threatened by the known toxic effects of contamination with trace elements, mostly in low- and middle-income countries. This is because precautionary measures to diminish contamination with trace elements are inadequate (Pure Earth 2015). On the contrary, the novel use of Ag is currently booming in high-income countries due to increasing applications in many aspects of daily life, commonly in the form of nanoparticles, with rarely known consequences for aquatic environments (Bruneau et al. 2016). There is nearly no control of Ag emissions into the environment, which may evolve into a serious problem even in high-income countries in the future.

Surveillance of trace elements in fish has two benefits. Fish have been recognized as bioindicators for environmental contamination, providing an integrated insight into the status of their environment over longer periods of time. This is particularly valid for most metals, as they show very long biological half-lives. Therefore, elevated tissue concentrations can occur even if the exposure is not continuous (Hofer et al. 1995). Moreover, fish is an important food source for humans in many parts of the world, and monitoring their trace element levels is therefore also important to ensure food safety. This is especially the case for Hg because fish consumption is believed to be the main source of this element for humans (McKelvey et al. 2012). Numerous studies have evaluated trace element levels in fish since awareness of environmental pollution awoke in the late 1960s and early 1970s (e.g., Reichenbach-Klinke 1974 and references therein), mostly from waterbodies in Europe and North America. Data from the African continent are scarce, and only little research has dealt with trace metal levels in fish (e.g., Avenant-Oldewage and Marx 2000; Mutia et al. 2012; Otachi et al. 2014, 2015; Lynch et al. 2016).

Lake Turkana and Lake Naivasha are two important waterbodies in the Kenyan Rift Valley. Lake Turkana is one of the largest freshwater bodies in Africa and is believed to be pristine, while Lake Naivasha is a small lake heavily influenced by anthropogenic activities within the lake and its surroundings. They differ considerably in their physicochemical properties. Lake Turkana is a very large, slightly saline lake (EC = 2000–3000 μS/cm (Olago and Odada 1996)) with very few human activities in the surrounding areas. Nevertheless, the lake’s ecosystem is poised for a potentially enormous change due to the ongoing construction of Gibe III dam in Ethiopia, the second largest hydroelectric power plant in Africa. This project will dam the Omo River in Ethiopia, the major source of water for the lake, and the consequences for the lake’s properties remain unknown. In contrast, Lake Naivasha is a much smaller lake containing freshwater (EC = 250–430 μS/cm (Otachi et al. 2014)). The surrounding areas as well as the lake itself are highly influenced by human activity, mostly floriculture and geothermal power generation. Both lakes are important sources of protein for the local population in the form of fish. This makes monitoring trace elements very important with regard to food safety. Otachi et al. (2015) delivered the first comprehensive study on trace element levels from water, sediment, and one fish species from the central part of Lake Turkana. Data from Lake Naivasha have been published by Tarras-Wahlberg et al. (2002), Ochieng et al. (2009), Kamau et al. (2008), and others who have already investigated the levels and sources of metals such as Cu, Cd, Zn, Fe, Pb, and Ni in Lake Naivasha. In addition, Njogu et al. (2011) and Mutia et al. (2012) showed that the most important sources of metal pollution in Lake Naivasha basin are its major tributary, the Malewa River, and some flower farms surrounding the lake. All these studies had a limited spectrum of investigated elements. Otachi et al. (2014) were the first to include a variety of trace elements in sediments and the Blue belly tilapia *Oreochromis leucostictus*. All these studies revealed elevated levels of trace elements, calling for further investigations: For Lake Turkana, the report by Otachi et al. (2015) showed elevated levels for some trace elements in the elongate tigerfish *Hydrocynus forskahlii* although no elevated levels in the sediment or water were observed from their sampling area in the central region of the lake. The authors hypothesized that *H. forskahlii* might be exposed to high contaminant levels during its annual migration into the Omo River, which drains a large basin used for agricultural purposes. The assumption is that the water is polluted with, amongst others, contaminants that commonly are found in fertilizers, such as Li and Cd (Otachi et al. 2015). Nonetheless, it remained unclear whether the findings for the central part of the lake regarding water and sediment were accidental or if they can be confirmed using a stationary fish species as bioindicator, i.e., by a species that does not migrate during its lifetime and therefore gives an integrated depiction of the trace element situation of its habitat. As a follow-up to the study of Otachi et al. (2015), we chose the red belly tilapia *Tilapia zillii* from the same sample area to gain further insight into a possible trace element contamination of central Lake Turkana.

In addition, we compared trace element levels in *T. zillii* caught in 2011 in Lake Naivasha in this study. The water levels in Lake Naivasha rose drastically in recent years, leading to a change in the physicochemical properties of the lake. To determine the consequences of this change in water level to the trace element accumulation in fish, we analyzed the metal concentration in *T. zillii* and in the common carp *Cyprinus carpio*, both caught in 2015. These values are compared with those measured in *T. zillii* collected in 2011 and in *C. carpio*, whose data were published by Mutia et al. (2012) (samples taken in 2010).

In summary, this study pursued three major aims: the determination of the differences in trace element levels between a migratory and a stationary fish species in Lake Turkana, the comparison of trace element levels in *T. zillii* from Lake Naivasha and Lake Turkana, and the temporal aspect of trace element accumulation in two fish species in Lake Naivasha between 2011 and 2015.

## Materials and methods

### Study site descriptions

**Fig. 1:**
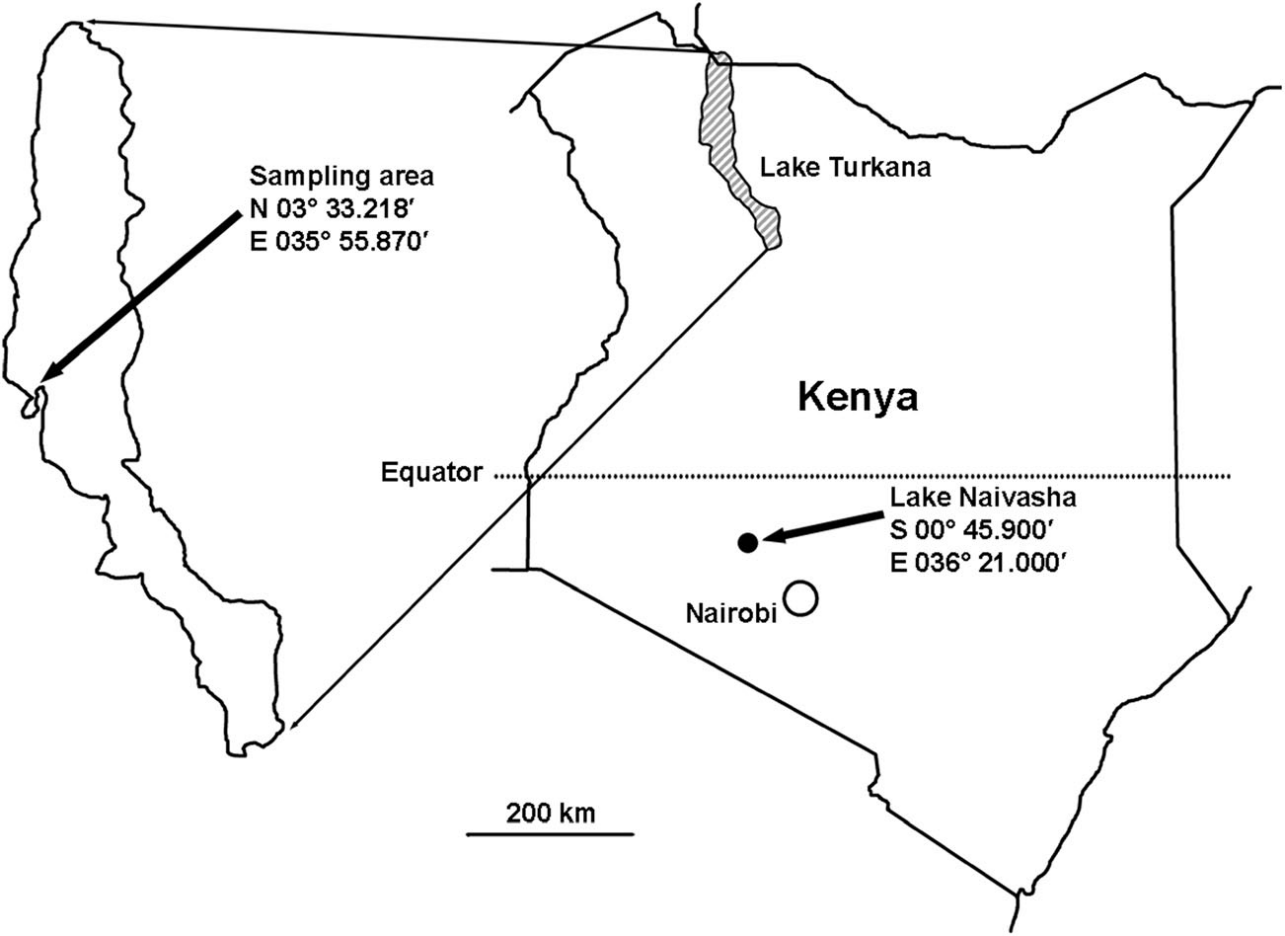
Geographical locations of the two study sites in Kenya

Lake Turkana (formerly Lake Rudolf) is located at the north of the Eastern Rift Valley in Kenya. It stretches from 35° 50′ to 36° 40′ E and 2° 27′ to 4° 40′ N, with its northernmost tip extending into Ethiopia (Fig. 1). It is the largest (7560 km^2^) and deepest water mass situated almost wholly in Kenya, with a maximum depth of 115 m and a mean depth of 30 m (Getabu et al. 2007). The lake is situated in an arid and hot environment, is slightly alkaline, and displays a specific conductivity of 2000–3000 μS/cm (Yuretich and Cerling 1983; Olago and Odada 1996). The human population density in the lake basin is very low (1–3/km^2^) (Odada et al. 2003). Lake Turkana receives 90% of its water from Omo River in Ethiopia and seasonally from Turkwell and Kerio Rivers in Kenya. The catchment area is approximately 130,860 km^2^, and the land use is dominated by pasture (47.5%), herbaceous vegetation (45%), and wood vegetation (5%), while crop fields cover 2.4% (ILEC 2013). For this study, fish were collected at the west bank of the lake at N 03° 33.218′ E 035° 55.870′.

Lake Naivasha is situated at 00° 45′ S and 36° 20′ E in a closed basin at an altitude of 1890 m above sea level in the Eastern Rift Valley of Kenya (Kamau et al. 2008). The lake covers approximately 160 km^2^. It is the only freshwater lake in the Rift Valley without a surface outlet but with a substantial exchange with groundwater (Clarke et al. 1990). It is shallow (approximately 6-m mean depth), with a volume of 4.6 km^3^. It is bordered by papyrus *Cyperus papyrus* in some sections, and the overall composition of aquatic macrophytes is in a state of change (Tarras-Wahlberg et al. 2002), probably due to anthropogenic influences such as destruction of littoral vegetation, eutrophication along with plant, and animal introductions. Most of its freshwater inflow (approximately 80–90%) comes from the Malewa River (Kamau et al. 2008) with an estimated mean annual flow of 153 million m^3^ and a catchment area of 1730 km^2^, followed by Gilgil River with an estimated average annual flow of 24 million m^3^ and a catchment area of 420 km^2^; an additional river, Karati River, flows only intermittently (Everard et al. 2002). The basin area is generally semiarid, receiving a mean annual rainfall of 620 mm, while the mean annual evaporation is estimated at 1735 mm. Evaporation generally exceeds precipitation throughout the year except at peak rainfall, with the rainfall trend being bimodal with a major peak in April–May and a minor peak in October–November. The water level in the lake was known to highly alternate in the past, although the intense abduction of water for irrigation and geothermal power generation led to decreasing mean levels (Harper et al. 2011). Since 2011, abundant rainfalls have led to an enormous increase in the water level again (Kuhn et al. 2016), resulting in a multiplication of the volume of the shallow lake and causing substantial changes in its water chemistry. For example, the mean pH was reduced from 9.0 (Otachi et al. 2014) in 2011 to 7.5 in 2014 (Johnson 2015); the mean specific conductivity decreased from 355 μS/cm in 2011 (Otachi et al. 2014) to 256 μS/cm in 2014 (Johnson 2015).

### Fish sampling

Fifteen specimens of the red belly tilapia *T. zillii* from Lake Turkana caught in 2011 and two sets of samples of ten specimens each from Lake Naivasha, one set caught in 2011 and another one in 2015, were analyzed. Fish were caught by fishermen using gill nets. Fish from Lake Turkana were kept in aerated tanks and transported alive to a laboratory at the Kenya Marine Fisheries Research Institute (KMFRI), whereas fish from Lake Naivasha were transported in the same manner to the laboratory at the Biological Sciences Department of Egerton University. In the respective laboratories, fish were killed by cervical dislocation and dissected. Ten specimens of *C. carpio* were obtained from the fish market at the banks of Lake Naivasha in 2015 and transported refrigerated to the laboratory at the Biological Sciences Department of Egerton University. Tissue samples of all fish (approximately 1 g of dorso-caudal muscle and the identical mass of liver) were obtained using a ceramic knife and plastic tweezers. After drying at 80 °C until constant weight, the samples were sent to the University of Vienna, Institute of Inorganic Chemistry, for further analyses.

### Trace element analysis

Sample preparation was done as described before (Otachi et al. 2014, 2015). Briefly, 0.2 g of dry sample was digested in 9 ml of HNO_3_ 34% (TraceSELECT® Fluka, Steinheim, Germany) in PTFE tubes using a microwave-assisted digestion system (MARS XPRESS by CEM Corporation, Matthews, USA). Digested samples were transferred into volumetric flasks and brought to volume with Millipore water. Samples were filtered through 0.2-μm PTFE syringe filters (VWR, Radnor, USA) and stored in polypropylene (PP) tubes. For the measurement, the samples were diluted with Millipore water where necessary. In addition, reference samples were prepared by treating 0.2 g (dry weight) of fish protein DORM-3 obtained from the National Research Council Canada (NRCC, Ottawa, Canada) in the same manner as described above for fish tissues. For the determination of the detection limits, analytical blanks were prepared without insertion of a sample. Li, Al, Cu, and Rb were determined using inductively coupled plasma-optical emission spectrometry (ICP-OES) using an Optima 5300DV (PerkinElmer, Waltham, USA); Zn was measured with flame atomic absorption spectrometry (F-AAS) using an AAnalyst 200 (PerkinElmer). For Cr, Ag, Cd, and Pb, graphite furnace atomic absorption spectrometry (GF-AAS) using a PinAAcle 900Z (PerkinElmer) was applied to obtain lower limits of detection. The limits of detection (all given in mg/kg dry weight (dw)) for elements in tissue were 0.005 for Cd; 0.007 for Cr, Cu, and Pb; 0.015 for Ag; 0.75 for Li and Rb; and 5.0 for Al and Zn. The results for the reference samples showed recovery rates between 95 and 104%, demonstrating the appropriateness of the sample preparation used.

Statistical analysis was done using IBM SPSS 22. The Kolmogorov-Smirnov test was used to check for normal distribution. Homogeneity of variances was tested using the nonparametric Levene’s test. As data were not normally distributed for all of the elements and homogeneity of variances was only given for Cu (*p* > 0.05), the nonparametric Mann-Whitney *U* test was used. For comparison, we multiplied wet weight data from literature (e.g., Mutia et al. (2012)) by a factor of 5 to refer to dry weight and calculated means and standard deviation. All concentrations presented in the text refer to dw.

## Results and discussion

Element concentrations in muscle and liver are presented in Tables 1 and 2, respectively. For *T. zillii* from Lake Naivasha from 2011, no levels of Li and Zn are available because this set of samples was initially part of a different survey, and therefore, the two elements mentioned were not measured. In the following section, trace elements are discussed in brief:

- Li has rarely been analyzed in fish; in particular, reports from freshwater species are scarce. For marine species, Li values from fish muscle reported in literature vary over three orders of magnitude, e.g., from 0.18 ± 0.03 mg/kg dw in various muscle tissues from a ling *Genypterus blacodes* off New Zealand (Ashoka et al. 2011) to 231.5 mg/kg fw in tuna from the northern Pacific Ocean (Hansen et al. 1978). Otachi et al. (2015) reported high concentrations of 206 ± 87.4 mg/kg dw in *H. forskahlii* from Lake Turkana, but levels were below the LOD of 0.75 mg/kg dw in all samples from this survey, including *T. zillii* from Lake Turkana. The source of Li in *H. forskahlii* is still unknown, but contact must have occurred in a different environment. Due to the chemical similarities of Li to Ca and Mg (Holleman and Wiberg 1976), precipitation of Li is expected even if there would be a source of Li to the lake (e.g., rivers polluted with Li from mineral fertilizers) in the river delta, as it has been described for Mg and Ca (Yuretich and Cerling 1983). Therefore, Li would hardly be bioavailable in the region under investigation in the central area of the lake (see also the explanations given for Cd below).
- Al is the most prevalent metal in the earth’s crust (Bowen 1979) but has no known biological function. Nonetheless, it is present in most biological samples. Except for *C. carpio*, Al levels were higher in liver compared to muscles in all samples, which conforms to the literature: e.g., Moiseenko and Kudryavtseva (2001) reported Al levels in white fish *Coregonus lavaretus* from the polluted Kola region of Russia (4.8 ± 0.6 μg/g dw in muscle and 19 ± 2.9μg/g dw in liver); even higher Al values were reported by Budambula and Mwachiro (2006) for redeye labeo *Labeo cylindricus* from the polluted Nairobi River, Kenya (70.0 μg/g wet weight (ww) for muscle and 150 μg/g ww for liver). Mean Al levels in muscle in our survey were between <5.0 mg/kg dw for *T. zillii* from Lake Turkana (from 2011) and 23.3 mg/kg dw in *C. carpio* from Lake Naivasha in 2015 and therefore compare well with the literature. In liver, mean levels ranged from 9.60 mg/kg dw in *T. zillii* from Lake Turkana in 2011 to 58.3 mg/kg in *T. zillii* from Lake Naivasha in 2015. Comparing *T. zillii* from the two lakes, those from Lake Turkana showed lower Al levels in both tissues. This is a further indicator for the pristine status of the central part of Lake Turkana. In *T. zillii* from Lake Naivasha, no significant differences in Al level between samples from 2011 to 2015 were observed in muscle and liver, respectively.
- Cr was detectable in all samples. Although Vincent (2001) concluded in his review that the element appears to be a trace nutrient for mammals, its essentiality for plants and animals is still under discussion (Markert et al. 2015). Except for *C. carpio*, Cr levels were higher in liver compared to muscles in all samples, in agreement with the literature. For example, Lynch et al. (2016) reported 0.22 mg/kg dw in muscle and 0.41 mg/kg dw in liver of *Labeo capensis* from the Vaal Dam reservoir in South Africa. Comparing *T. zillii* from the two lakes (2011), significant differences occurred in muscle (0.42 mg/kg dw in Lake Naivasha; 0.23 mg/kg dw in Lake Turkana), but not in liver. Comparing samples from 2011 to 2015, in *T. zillii* from Lake Naivasha, again a clear decrease of Cr levels is evident in both muscle and liver.
- Cu levels were higher in liver than in muscle in all samples, which generally agrees with many other findings (Papagiannis et al. 2004, Jirsa et al. 2008, Lynch et al. 2016). This reflects the livers’ important role in storing and detoxifying Cu. Levels in liver do not differ significantly between the different sampling points and sampling times. In muscle, the levels are higher in all samples from Lake Naivasha (*T. zillii* and *C. carpio*) than in those from Lake Turkana (*T. zillii* and *H. forskahlii*). Considering the temporal aspect in Lake Naivasha, no significant change of Cu levels in muscle of *T. zillii* occurred between 2011 and 2015. In *C. carpio* from Lake Naivasha, our levels in muscle from 2015 (1.12 mg/kg dw) are lower than those reported by Mutia et al. (2012) from 2010, which might reflect the change in the lake’s water chemistry.
- Zn levels in liver were higher than in muscle. This agrees with most of the literature on Zn in freshwater fish (Al-Yousuf et al. 2000; Jirsa et al. 2008; Lynch et al. 2016) but contrasts with previous studies on element concentrations in *O. leucostictus* from Lake Naivasha and *H. forskahlii* from Lake Turkana (Otachi et al. 2014, 2015).

**Table 1:** Trace elements in muscle tissues

	*T. zillii* 2011	*H. forskahlii* 2011	*T. zillii* 2011	*T. zillii* 2015	*C. carpio* 2015	*C. carpio* 2010
	L. Turkana	L. Turkana^1^	L. Naivasha	L. Naivasha	L. Naivasha	L. Naivasha^2^
Li	<0.75	206 ± 87.4	n.m.	<0.75	<0.75	n.m.
Al	<5.0	6.71 ± 2.9	5.24 ± 8.83^a^	9.10 ± 1.68^a^	23.3 ± 4.7^b^	n.m.
Cr	0.232 ± 0.246^a^	n.m.	0.42 ± 0.33^b^	0.075 ± 0.019^c^	0.120 ± 0.184^c^	n.m.
Cu	0.808 ± 0.219^a^	0.63 ± 0.46	1.23 ± 1.41^b,c^	1.70 ± 0.29^c^	1.12 ± 0.28^b^	5.70 ± 5.30
Zn	26.9 ± 5.7^a^	426 ± 215	n.m.	23.9 ± 4.5^a^	32.8 ± 33.4^a^	n.m.
Rb	32.3 ± 63.3^a,b^	5.84 ± 2.85	53.8 ± 102^b^	14.9 ± 2.4^a,b^	19.6 ± 6.3^a^	n.m.
Ag	<0.015	n.m.	<0.015	<0.015	<0.015	n.m.
Cd	<0.005	0.56 ± 0.35	0.038 ± 0.03	<0.005	<0.005	6.75 ± 1.40
Pb	<0.007	0.012 ± 0.002	<0.007	<0.007	<0.007	165 ± 92

All values in milligram per kilogram dw ± standard deviation

*n.m.* element not measured in the sample. Same letters in a row indicate no significant difference (*p* < 0.05)

^1^Data from Otachi et al. (2015), not included in comparative statistical analysis

^2^Data from Mutia et al. (2012), not included in comparative statistical analysis. Original values from Mutia et al. (2012) are multiplied by a factor of 5 to estimate milligram per kilogram dw

**Table 2:** Trace elements in liver tissues

	*T. zillii* 2011	*H. forskahlii* 2011	*T. zillii* 2011	*T. zillii* 2015	*C. carpio* 2015
	L. Turkana	L. Turkana^1^	L. Naivasha	L. Naivasha	L. Naivasha
Li	<0.75	<0.75	n.m.	<0.75	<0.75
Al	9.60 ± 4.63 ^a^	18.0 ± 11.2	17.6 ± 31.1 ^b^	58.3 ± 42.6^bc^	16.2 ± 6.1^c^
Cr	1.07 ± 0.47 ^a^	n.m.	1.65 ± 1.24 ^a^	0.351 ± 0.234^b^	0.03 ± 0.01^c^
Cu	96.8 ± 100.4 ^a^	17.2 ± 6.05	123 ± 148^a^	26.4 ± 20.5^a^	28.4 ± 22^a^
Zn	75.5 ± 28.2 ^a^	89.2 ± 21.1	n.m.	67.7 ± 7.8^a^	570 ± 757^b^
Rb	26.2 ± 45.8^a^	<0.75	32.3 ± 51.1^a^	6.95 ± 2.80^a^	8.62 ± 5.99^a^
Ag	0.243 ± 0.199 ^ab^	n.m.	0.427 ± 0.359 ^ab^	0.200 ± 0.143^a^	0.062 ± 0.055^b^
Cd	0.255 ± 0.191 ^a^	11.5 ± 6.74	0.577 ± 0.445^b^	0.030 ± 0.018^c^	0.091 ± 0.171^c^
Pb	<0.007	0.015 ± 0.004	<0.007	0.531 ± 0.377	<0.007

All values in milligram per kilogram dw ± standard deviation. Same letters in a row indicate no significant difference (*p* < 0.05)

*n.m.* element not measured in the sample

^1^Data from Otachi et al.(2015) not included in comparative statistical analysis

In muscle of *T. zillii* from Lake Turkana, a mean Zn content of 26.9 mg/kg dw was measured. This is much lower than the mean level of 426 mg/kg dw reported in muscle of *H. forskahlii* from the same sampling site by Otachi et al. (2015). Comparing the mean levels in liver of the two fish species from Lake Turkana, however, did not show significant differences (75.5 ± 28.2 mg/kg dw for *T. zillii* and 89.2 ± 21.1 mg/kg dw *H. forskahlii*, respectively). A possible explanation for the higher Zn levels in *H. forskahlii* from Lake Turkana is given below.

Mean Zn contents in muscle of *T. zillii* from Lake Turkana and from Lake Naivasha did not show significant differences (26.9 ± 5.7 mg/kg dw from Lake Turkana (2011) and 23.9 ± 4.5 mg/kg dw from Lake Naivasha (2015), respectively) and compare very well with the literature, relatively independent of the fish species being compared. For example, Amundsen et al. (1997) found values of 17–63 mg/kg dw in muscle from seven different freshwater species from northern Norway, and Lynch et al. (2016) reported a mean Zn level of 29.4 mg/kg dw in muscle of *L. capensis* from the Vaal Dam reservoir in South Africa. The Zn levels found in this study in muscle of *T. zillii* stand in contrast to the mean Zn content of 426 ± 215 mg/kg dw found by Otachi et al. (2014) in muscle of *O. leucostictus* sampled in 2011 from Lake Naivasha.

Levels in liver of *T. zillii* of 75.5 ± 28.2 mg/kg dw from Lake Turkana (2011) and 67.7 ± 7.8 mg/kg dw from Lake Naivasha (2015) compare well with the previous studies by Otachi et al. (2014, 2015), who found mean levels of 73.1 mg/kg dw in liver of *O. leucostictus* from Lake Naivasha and 89.2 mg/kg dw in liver of *H. forskahlii* from Lake Turkana. Compared to the literature, the levels that we report in liver of *T. zillii* from both lakes are on the lower end of the scale. For example, Lynch et al. (2016) reported a mean of 179.3 mg/kg dw in liver of *L. capensis* from the Vaal Dam. Moiseenko et al. (2001) found mean levels between 121 and 163 mg/kg dw in liver of *C. lavaretus* and *Salmo trutta* from areas affected by mining and metallurgical enterprises in the Kola region in Russia.

The Zn levels in muscle of *C. carpio* from Lake Naivasha of 32.8 ± 33.4 mg/kg dw compare well with those for *T. zillii*, as well as with the literature mentioned above. Similar values around 20 mg/kg dw have been reported for *C. carpio* from a Greek freshwater lake by Papagiannis et al. (2004). The levels in liver of *C. carpio* from Lake Naivasha were significantly higher compared to *T. zillii* from this study, but are still in accordance with Zn levels reported by, e.g., Amundsen et al. (1997) for *Coregonus albula* from the border region between Norway and Russia.

The high difference in Zn levels between muscle and liver in *C. carpio* from Lake Naivasha also supports the theory of Otachi et al. (2015) that elevated Cd levels could also be responsible for an enhanced Zn content in muscle. As an essential trace element, the uptake of Zn is highly regulated by the organism (Bury et al. 2003). Cd blocks Zn-containing enzymes, enhancing their expression and possibly leading to elevated Zn levels (Kopera et al. 2004). The high levels of Cd in *H. forskahlii* from Lake Turkana as well as in *O. leucostictus* from Lake Naivasha (Otachi et al. 2014) could have led to an enhanced Zn uptake into muscle of those fish. Due to the low Cd levels in muscle and liver of *C. carpio* from Lake Naivasha, the correct regulation of the Zn uptake into muscle could have been possible. Therefore, no elevated Zn levels could be observed in muscle of *C. carpio*, although the levels in liver were higher than in the other species in our survey.

We found highly variable concentrations of Rb in *T. zillii* muscle from all sample groups as well as in carp muscle, but the values were not significantly different from each other. Mean levels were between 14.9 and 53.8 mg/kg dw. These values are well comparable to those reported by Otachi et al. (2014) for *O. leucostictus* from Lake Naivasha (mean 18.2 mg/kg dw). They also agree well with values reported by Silva and Shimizu (2004) in the flesh of nine fish species in Sri Lanka (20.90–70.75 mg/kg dw), including the relatively closely related cichlid species *Oreochromis mossambicus*, *Oreochromis niloticus*, and *Tilapia rendalli*. Compared to Rb levels from *T. zillii*, the concentrations found in *H. forskahlii* (5.84 ± 2.85 mg/kg dw) from Lake Turkana by Otachi et al. (2015) are much lower. This result stands in a certain contrast to the biomagnification of Rb in freshwater food webs postulated by Campbell et al. (2005), as *H. forskahlii* is one of the top predators in Lake Turkana (trophic level 4.0 ± 0.75) and *T. zillii* ranges well below that (trophic level 2.5 ± 0.1) (Froese and Pauly 2017).

We found Rb in both muscle and liver of *T. zillii* and *C. carpio*, showing no significant differences between these two tissues. This agrees with the findings of Underwood (1971) that Rb is not accumulated in any specific organ or tissue. Literature on Rb levels in liver is scarce, but the levels that we report compare well to those presented by Otachi et al. (2014) for *O. leucostictus* from Lake Naivasha (mean 15.6 mg/kg dw).

Although Rb has not been a major topic in this field, we propose to enhance studies on this element in the future: experimental toxic responses linked with elevated Rb have been observed in mammals fed low-K high-Rb diets; these responses are probably linked to physiological interference with K and Na (Kosla et al. 2002).

Ionic Ag has been known to be highly toxic to fish for decades (Davies et al. 1978; Hogstrand and Wood 1998), but some studies indicate that Ag nanoparticles may have toxic effects on aquatic life in even lower concentrations than ionic Ag (Fabrega et al. 2011). In this study, Ag concentrations in muscle were below the limit of detection of 0.015 mg/kg dw for all samples.

Results for Ag levels in liver of *T. zillii* and *C. carpio* were well comparable. In liver of *T. zillii* from Lake Turkana, we measured a mean level of 0.243 mg/kg dw and in those from Lake Naivasha 0.427 mg/kg dw (2011) and 0.200 mg/kg dw (2015). The mean level in liver of common carp from Lake Naivasha was 0.060 mg/kg dw. Literature on Ag levels in freshwater fish is scarce. Yamazaki et al. (1996) reported a value of 1.35 mg/kg Ag in liver of a single individual of *Carasius auratus langsdorfii* from a river in Tokyo, Japan. Ahmed et al. (2016) reported Ag levels in liver of *C. carpio* and four other fish species from Pakistan between 69.7 and 74.6 mg/kg dw (converted from ww to dw by multiplying with a factor of 5). Accordingly, the levels found in our study are about two to three orders of magnitude lower than these reported by Ahmed et al. (2016). We assume that the “silver boom” has not reached the African continent; therefore, these levels might represent background levels and will be of importance for future comparison.

Cd levels in liver were higher than in muscle, which generally agrees with the literature on freshwater fish (Jirsa et al. 2008; Al-Kenawy and Aly 2015; Otachi et al. 2015; Arantes et al. 2016).

Cd content in muscle of *T. zillii* from Lake Turkana was below the limit of detection of 0.005 mg/kg dw. In liver, the mean concentration was 0.255 ± 0.191 mg/kg dw. These levels are much lower than in the previous study on element concentrations in *H. forskahlii* from Lake Turkana, where Cd concentrations of 0.56 ± 0.35 mg/kg dw in muscle and 11.5 ± 6.74 mg/kg dw in liver have been measured (Otachi et al. 2015). A possible explanation for this big difference in Cd levels between the two species from Lake Turkana, which were sampled at the same site and the same time, might be the annual migration of *H. forskahlii* into the highly influenced Omo River (Kolding 1989). Trace element data from the river are missing, but the water is no doubt polluted with, amongst others, contaminants that are common in mineral fertilizers, such as Cd (Alloway and Steinnes 1999; Dissanayake and Chandrajith 2009). Due to the high carbonate content in the lake (Odada et al. 2003), precipitation of cadmium is conceivable in the river delta of the Omo River, as has been described in the past for magnesium and calcium (Yuretich and Cerling 1983). This would remove most of a possibly high cadmium freight of the Omo River in the mixing zone. Therefore, the nonmigratory *T. zillii* from the central area of the lake, which stays within the same region of the lake its entire life, is not exposed to elevated Cd levels (Otachi et al. 2015). This interpretation is also strengthened by the low Cd content in the sediment at the west bank of the lake, whereas *H. forskahlii* might get in contact with soluble Cd salts during its annual migration into the Omo River (Otachi et al. 2015). Like many other metals, Cd shows a very long biological half-life in fish (Hofer et al. 1995). If fish pass through waters with easily soluble salts of these elements, accumulation can occur even if the exposure is not continuous.

In muscle of *T. zillii* from Lake Naivasha (2011), 0.038 ± 0.35 mg/kg Cd was measured, while Cd levels were below the limit of detection (< 0.005 mg/kg) in muscle of *T. zillii* from Lake Turkana (2011). Levels in liver were 0.577 ± 0.445 mg/kg in fish from Lake Naivasha and 0.255 ± 0.191 mg/kg dw in those from Lake Turkana. Therefore, in 2011, Cd levels in both liver and muscle were higher in *T. zillii* from Lake Naivasha than in those from Lake Turkana. This agrees with the higher anthropogenic impact in the areas surrounding Lake Naivasha versus Lake Turkana.

Considering the temporal aspect between 2010/2011 and 2015 in Lake Naivasha, we observed a clear decrease of Cd levels in *T. zillii* and *C. carpio*. A mean concentration of 0.031 mg/kg in liver of *T. zillii* was measured in the samples from Lake Naivasha from 2015, which is on the lower end of Cd levels published for freshwater fish (Jirsa et al. 2008, Jarić et al. 2011, Otachi et al. 2014). For example, Ogamba et al. (2016) reported similar low Cd levels in liver of *Clarias garepinus* from the Niger Delta. The levels in muscle were below the limit of detection in *T. zillii* from 2015. These values in muscle as well as in liver are much lower than those from 2011. The same trend occurred for *C. carpio*. The levels that we measured in muscle of *C. carpio* from Lake Naivasha were below the limit of detection of 0.005 mg/kg. This is much lower than the values reported by Mutia et al. (2012): 6.75 mg/kg dw in muscle of *C. carpio* from Lake Naivasha. This supports the theory of a decreasing level of pollution with heavy metals in Lake Naivasha during recent years. This decrease may be caused by the above-mentioned enormous increase of the water level and the change in the water chemistry of Lake Naivasha. Nevertheless, values presented by Mutia et al. (2012) may have to be considered with caution as they seem to be unlikely high. To our knowledge, no studies link the Cd concentration in muscle tissue and toxic effects in *C. carpio*, but Handy (1993) reported that Cd levels in muscle as low as 0.35–1.7 mg/kg were connected with death of rainbow trout *Oncorhynchus mykiss*. Although this comparison should be made with caution because *C. carpio* is known to be much more tolerant towards Cd than *O. mykiss* (de Conto Cinier et al. 1999), at least severe sublethal effects should have occurred in these carps, which were not reported.

*T. zillii* from 2015 from Lake Naivasha showed a mean Pb level in liver of 0.531 ± 0.377 mg/kg dw, while the level in muscle was below the limit of detection of 0.07 mg/kg dw. Levels in *T. zillii* from 2011 from both lakes as well as *C. carpio* from Lake Naivasha (2015) were below the detection limit in both muscle and liver.

These rather low Pb concentrations compare well with our previous studies on fish from the two lakes as well as with the low levels of Pb in the lake sediments (Otachi et al. 2014, 2015). In contrast, Mutia et al. (2012) reported a mean Pb level of 165 mg/kg dw in *C. carpio* from Lake Naivasha. This level seems extremely high, and to our knowledge, it is the highest Pb value ever reported in freshwater fish. Muscle tissue is not a primary target for the storage of Pb. Even experiments with long-term exposure to foodborne Pb in *Carassius gibelio*, a fish from the cyprinid family, like *C. carpio*, led to a mean content in muscle of 3.6 mg/kg dw (Łuszczek-Trojnar et al. 2013); this obvious discrepancy might again indicate that values presented by Mutia et al. (2012) should be considered with caution.

## Conclusions

For most trace elements, liver accumulated higher levels than muscle. In contrast to previous work on *H. forskahlii*, we did not detect high levels of Cd in *T. zillii* from Lake Turkana, indicating that *H. forskahlii* does not become exposed to high Cd levels during its stay in the region under investigation. Most trace elements show very long biological half-lives (Hofer et al. 1995). Our data therefore allow the conclusion that—at least during the lifetime of the sampled fish—there was no contamination in their habitat. Trace element levels in *T. zillii* from Lake Turkana confirm the pristine state of the central region regarding the elements under investigation, at least for the years 2009–2011, as fish sampled in 2011 were estimated to be 3 to 4 years old. We furthermore conclude that using fish as bioindicators is a valuable tool when the lifestyle of the fish species is known. Analysis of water and sediment gives only a snapshot of the conditions of the lake, while fish give an integrated view over their lifetime due to the long half-life of many pollutants. *T. zillii*, which spends its whole life in the same region of the lake, provided a much better overview of the local conditions at the sampling site than the migratory *H. forskahlii*.

The rare opportunity to compare one fish species, namely *T. zillii*, from contrasting lakes demonstrates that fish deal differently with different types of trace elements: physiologically regulated and essential elements such as Cu and Cr appear in a very similar range in fish organs from the two lakes, showing the regulatory ability of the fish species under various water conditions, namely significantly different EC, pH, and major ion content. In contrast, levels of nonessential metals such as Cd, Pb, and Ag in fish rather reflect short- or long-term exposure to these elements. For *T. zillii* from Lake Naivasha, we report lower concentrations of Rb, Cd, and Cr in 2015 compared to 2011. In *C. carpio* from 2015, we also measured much lower values for most trace elements than Mutia et al. (2012) in samples from 2010. This most probably reflects the massive change in the lake’s water level and water chemistry during the last years.
